# Freiburg Neuropathology Case Conference: A 42-year-old Patient with Progressive Neurological Deficits, Multiple Brain Lesions and Accompanying Affection of Peripheral Nerves

**DOI:** 10.1007/s00062-021-01021-5

**Published:** 2021-06-11

**Authors:** M. Frosch, N. Kremers, K. Lisko, H. Urbach, M. Prinz, C. A. Taschner

**Affiliations:** 1 Departments of Neuropathology, Medical Centre, University of Freiburg, Faculty of Medicine, University of Freiburg, Freiburg, Germany; 2 Departments of Neuroradiology, Medical Centre, University of Freiburg, Faculty of Medicine, University of Freiburg, Breisacherstr. 64, 79106 Freiburg, Germany; 3 Departments of Neurology and Neurophysiology, Medical Centre, University of Freiburg, Faculty of Medicine, University of Freiburg, Freiburg, Germany

**Keywords:** Susac syndrome, Anti-MOG associated encephalomyelitis, Central nervous system vasculitis, Multiple sclerosis, Guillain-Barré Syndrome

## Case Report

**Fig. 1:**
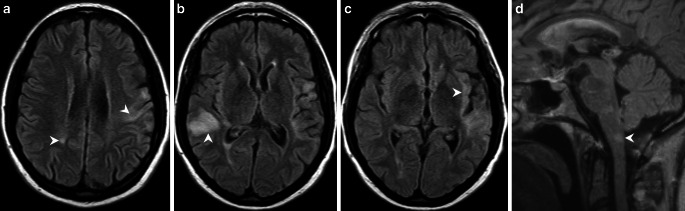
Axial fluid-attenuated inversion recovery (FLAIR) images show multiple hyperintense cortico-subcortical and white matter lesions. These lesions are located in the left precentral gyrus and the right-sided parietal white matter (**a**, *arrowheads*), the right superior temporal gyrus and angular gyrus (**b**, *arrowhead*), the left-sided insular cortex and subcortical areas (**c**, *arrowhead*), as well as dorsal parts of the medulla oblongata (**d**, *arrowhead*). The corpus callosum is not affected (**d**)

**Fig. 2:**
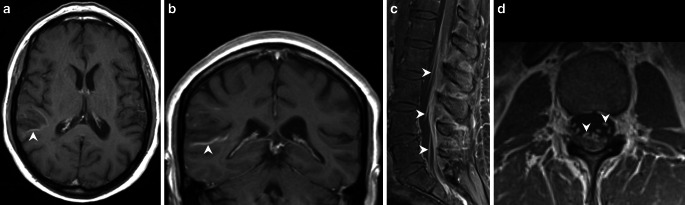
On axial (**a**) and coronal (**b**) T1-weighted images after administration of gadolinium the lesion located in the right temporal lobe shows slight rim enhancement (**a**, **b**, *arrowheads*). On sagittal (**c**) and axial (**d**) T1-weighted postcontrast images of the lumbar spine marked contrast enhancement of the spinal nerve roots in the cauda equina is visible (**c**, **d**, *arrowheads*). On-going chemoradiotherapy. The hyperintense signal intensity changes have markedly increased on axial T2-weighted images (**a**, *arrowhead*)

**Fig. 3:**
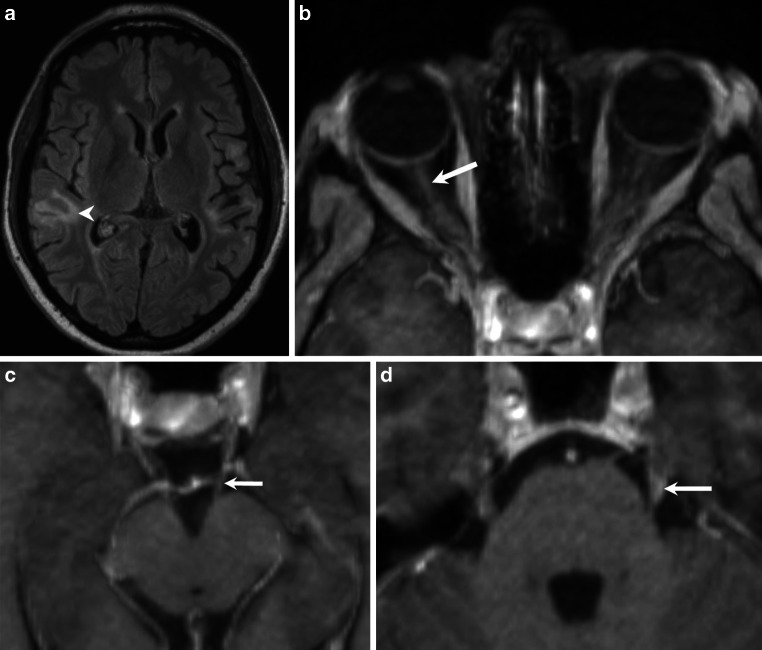
On axial FLAIR images (**a**) obtained 10 months later, the right-sided temporal lesion appears less pronounced (*arrowhead*). Axial T1-weighted postcontrast images (**b**–**d**) acquired during the same imaging session reveal contrast enhancement of the optic nerves (**b**, *arrow*), the oculomotor nerves (**c**, *arrow*), and the trigeminal nerves (**d**, *arrow*)

A 42-year-old patient presented 18 months ago with aphasia characterized by phonemic paraphasia. The patient additionally had a history of chronic alcohol abuse. The patient complained of progressive gait disorder 4 months later. Magnetic resonance imaging (MRI) of the brain at that time showed various white matter lesions, partially with a disrupted blood brain barrier (Figs. 1, 2a, b). The cerebrospinal fluid (CSF) showed an increased total amount of protein and a slightly increased lactate level. In addition, MRI of the lumbar spine revealed enhancement of the caudate nerves (Fig. 2c, d). The patient was diagnosed with a Guillain-Barré syndrome and received a course of intravenous (iv) immunoglobulins. The patient presented with a complete hearing loss and a blurred vision of the left eye 14 months later. The MRI showed supratentorial lesions (Fig. 3a) and a contrast medium enhancement of the optic nerves, oculomotor nerves, and the trigeminal nerves bilaterally (Fig. 3b–d). Furthermore, there was contrast enhancement of the meninges. The electrophysiological studies revealed a severe sensorimotor axonal demyelinating polyneuropathy. Fundoscopic examination revealed a faded optic nerve whereas signs of retinal vasculopathy were not present. Based on the presence of hearing loss, the MRI findings, and the results of the CSF analysis a small-vessel vasculitis, mostly likely Susac syndrome, was considered the most likely diagnosis. Under plasmapheresis for 5 days and steroid treatment the hearing loss decreased.

Due to the results of the ultrasound examination of peripheral nerves, which showed homogeneously thickened nerves and the extremely slowed nerve conduction velocity, an additional hereditary neuropathy (e.g. hereditary motor and sensory neuropathy type III, Dejerine-Sottas syndrome) was discussed. The muscle nerve biopsy of the sural nerve showed an end-stage neuropathy. After interdisciplinary discussion in the tumor board a combined stereotactic and leptomeningeal biopsy was performed.

## Differential Diagnosis, Clinical

### Susac Syndrome

Susac syndrome is an extremely rare vasculopathy with stenosis and obliteration of retinal and cerebral vessels probably caused by an autoimmune pathogenesis through complement accumulation and infiltration of lymphocytes. It presents with a clinical triad of encephalopathy (migraine-like headache, neuropsychiatric symptoms), retinopathy with vision loss and inner ear hearing loss. For the triad to appear simultaneously is relatively unusual. Treatment of Susac syndrome consists of a course of steroids (high-dose therapy + tapering), plasmapheresis, and iv immunoglobulins.

### Guillain-Barré Syndrome (GBS)

The acute or subacute, immune-mediated, polyradiculoneuritis is typically motor accentuated and a mostly symmetrically presenting polyneuropathy. Based on electrophysiological and pathological findings a demyelinating variant (acute inflammatory demyelinating polyneuropathy, AIDP) and an axonal variant (acute motor axonal neuropathy, AMAN) can be differentiated. Regarding the severe sensorimotor axonal, mainly demyelinating polyneuropathy we assume a chronic inflammatory demyelinating polyneuropathy (CIDP) due to the GBS. Multiple sclerosis and GBS are both autoimmune demyelinating disorders of central and peripheral nervous system. The coexistence of these two syndromes in an individual’s life span is rare and only reported in very few case reports.

### Atypical Presentation of Multiple Sclerosis (MS)

The patient suffered initially from an aphasia and a speech apraxia. During the following months, loss of hearing and vision were added. This would be an atypical presentation of MS. Sudden deafness has been reported in MS patients, in rare cases it can even be the initial manifestation. The link between MS and hearing loss is supposedly a result from a central impairment of audition, most likely at the level of the brainstem.

## Imaging

Initial MRI of the brain revealed multiple cortico-subcortical and white matter lesions, which appeared hyperintense on fluid attenuated inversion recovery (FLAIR) images. These lesions were located in the left precentral gyrus and the right-sided parietal white matter (Fig. 1a), the right superior temporal gyrus and angular gyrus (Fig. 1b), the left-sided insular cortex and subcortical areas (Fig. 1c) as well as dorsal parts of the medulla oblongata (Fig. 1d). The corpus callosum was not affected (Fig. 1d). After administration of gadolinium the lesion located in the right temporal lobe showed slight rim enhancement (Fig. 2a, b). Additional postcontrast images of the lumbar spine revealed marked contrast enhancement of the spinal nerve roots in the cauda equina (Fig. 2c, d). On FLAIR images obtained 10 months later the right-sided temporal lesion appeared less pronounced (Fig. 3a) and there were no signs of a disrupted blood-brain barrier on postcontrast T1-weighted images in this area (not shown). The T1-weighted postcontrast images revealed contrast enhancement of the optic nerves (Fig. 3b), the oculomotor nerves (Fig. 3c), and the trigeminal nerves (Fig. 3d). The vestibulocochlear nerves did not enhance (not shown).

## Differential Diagnosis, Imaging

### Multiple Sclerosis

Multiple sclerosis (MS) is a relatively common acquired chronic relapsing demyelinating disease involving the central nervous system and is the second most common cause of neurological impairment in young adults, after trauma [1]. Characteristically, and as defined by the McDonald criteria, multiple sclerosis is disseminated not only in space (i.e. multiple lesions in different regions of the brain) but also in time (i.e. lesions occur at different times) [2]. In our case dissemination in space and time criteria was met with supratentorial and infratentorial lesions as well as partly contrast-enhancing lesions and new lesions over time. A small peripheral lesion of the spine is especially a typical finding in MS. Atypical features were the predominantly juxtacortical distribution of lesions and very few pericallosal lesions. There were no “red flags” indicating to disregard the diagnosis (e.g. extensive changes in the spine, leptomeningeal enhancement). The enhancement of the cauda equina is also not typical for MS, although there are a few case reports with a co-occurrence of GBS and MS [3].

### Anti-MOG Associated Encephalomyelitis

Anti-MOG associated encephalomyelitis represents a group of inflammatory demyelinating disorders united by the presence of IgG antibodies to myelin oligodendrocyte glycoprotein (MOG) that overlap but are distinct from acute disseminated encephalomyelitis (ADEM), neuromyelitis optica spectrum disorder (NMOSD) and multiple sclerosis (MS). It typically affects children and young adults 30–40 years old [4]. As to this point, distinct imaging features of anti-MOG associated encephalomyelitis are not fully established. As in the clinical setting, there is an overlap with chronic demyelinating diseases and NMOSD. Generally, basal ganglia and infratentorial regions tend to be more affected and as in NMOSD the presence of a transverse myelitis (T2 signal abnormality in spinal cord ≥ 3 vertebral segments) or optic neuritis (especially the anterior part of optic nerve) is frequently reported. In addition, conus medullaris lesions are one of the imaging criteria [5]. Although there were no unequivocally typical intra-axial lesions (especially no classical transverse myelitis or optic neuritis), coexisting brain lesions with myelitis of the conus and cauda equina, may be a potential hint to this diagnosis and in the right clinical setting testing of MOG antibodies should be considered.

### Central Nervous System Vasculitis

Central nervous system (CNS) vasculitis or inflammatory and immunologically mediated vessel diseases (IIMVD) represent a heterogeneous group of inflammatory diseases affecting the walls of blood vessels in the brain, spinal cord, and the meninges. Imaging findings for primary angiitis of the CNS are usually variable and nonspecific, with ischemic infarctions the most common lesions. Possible findings also include lesions that might resemble MS, usually with the presence of associated findings that are unusual for MS (red flags), or that might resemble noninflammatory small vessel disease. Also encountered are focal, confluent, or diffuse T2 hyperintense lesions, mass-like lesions as well as meningeal enhancement, including presence of dural enhancement, epidural masses and leptomeningeal enhancement (homogeneous or nodular) and focal or extensive myelitis [6]. Small vessel vasculitis is always a differential diagnosis when imaging findings are complex and come in various synchronous but unspecific forms, as in the presented case. Although we did not see ischemic infarctions, leaving out the most typical element, secondary or (rarely) primary CNS vasculitis is a possible diagnosis.

### Susac Syndrome

Susac syndrome is a rare syndrome typically affecting young to middle-aged women that is clinically characterized by the triad of acute or subacute encephalopathy, bilateral sensorineural hearing loss, and branch retinal arterial occlusions. It is due to an autoimmune endothelialopathy leading to microinfarcts [7]. Diagnostic criteria have been proposed in 2016 that divide patients into definite and probable diagnosis of Susac syndrome based on the presence of certain clinical and imaging criteria. Imaging criteria for Susac syndrome are so-called snowball lesions centrally in the corpus callosum (round FLAIR hyperintense lesions), which are almost pathognomonic with at least one corpus callosum lesion present. In addition, leptomeningeal enhancement and well-demarcated T1 hypointense grey matter lesions with or without contrast enhancement support the diagnosis but are not mandatory [8]. Our case did not present any lesions matching the imaging criteria, making the diagnosis unlikely from an imaging point of view.

## Histology

**Fig. 4:**
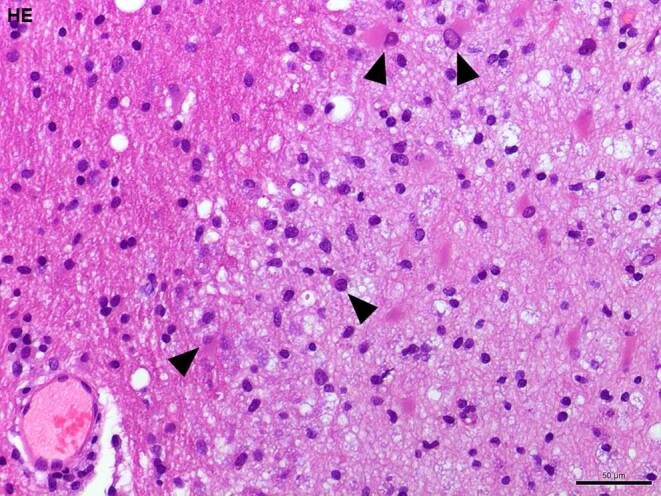
Hematoxylin-eosin (H&E) stained section showing gliotic altered white matter. *Arrowheads* indicate foam cells. Size bar 50 µm

**Fig. 5:**
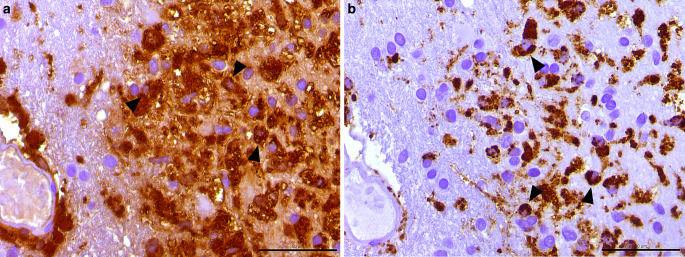
The immunohistochemical reactions against human leukocyte antigen (HLA)-DR (**a**) and the lysosomal activation marker cluster of differentiation (CD) 68 (**b**) show distinct positivity in the foamy altered macrophages (*arrowheads*). Size bar 50 µm

**Fig. 6:**
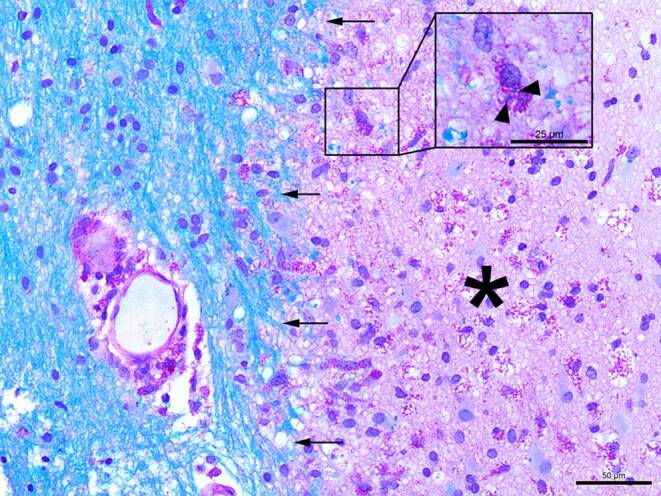
The luxol fast blue combined with periodic acid-Schiff (LFB-PAS) staining shows a clear-cut demyelinated lesion within the gliotic altered white matter indicated by the *arrows*. The demyelinated lesion itself is tagged with an *asterisk*. Size bar: 50 µm. Inlet: magnified section shows one foam cell with intracellular LFB-PAS positive debris (*arrowheads*). Size bar 25 µm

**Fig. 7:**
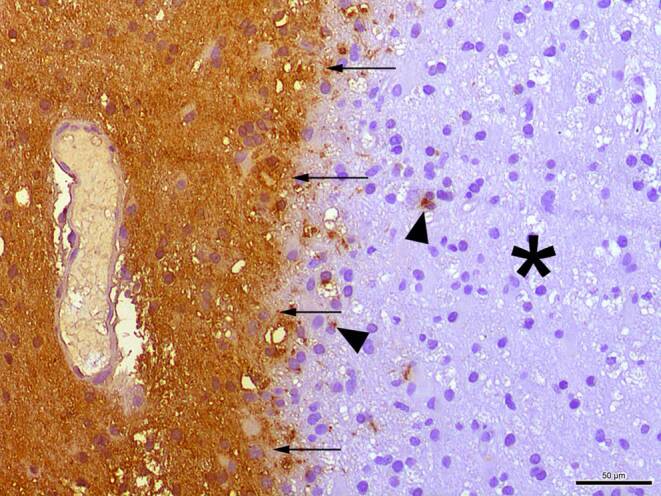
Immunohistochemical staining against myelin basic protein (MBP) reveals a sharply defined lesion margin (*arrows*). The *asterisk* marks the demyelinated area. *Arrowheads* indicate intracellular MBP inclusions. Size bar 50 µm

**Fig. 8:**
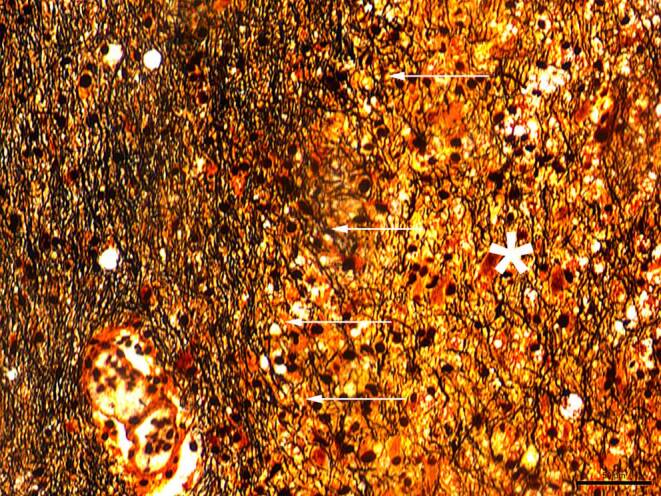
The Bielschowsky silver staining shows a rarefied axonal network within the lesion site marked with an *asterisk*. *Arrows* indicate the lesion border. Size bar 50 µm

In the hematoxylin-eosin (H&E) stained sections of the formaldehyde-fixed and paraffin-embedded stereotactic biopsy material, small fragments derived from the cortex and subcortical white matter were found, which both appeared gliotically altered. Within the white matter, sharply defined foci with many foam cells were conspicuous (Fig. 4). These cells showed an intense signal in the immunohistochemical staining for CD68 (Fig. 5a) and HLA-DR (Fig. 5b). In the luxol fast blue periodic acid Schiff (LFB-PAS) staining, these foci appeared completely faded, indicating the entire demyelination of these regions. The demyelination also exhibited a sharp border to the surrounding gliotically altered tissue (Fig. 6) and was observed in the immunohistochemical staining of myelin basic protein (MBP) as well (Fig. 7). Some of the foam cells contained PAS-positive and LFB-positive debris or MBP-positive myelin proteins, especially in the border areas (Figs. 6 and 7). Furthermore, regionally several lymphoid, perivascularly accentuated infiltrates were found, whereas distinct inflammatory infiltrates were missing (Fig. 4). The staining for CD3 defines these perivascular, infiltrating cells as T‑cells and showed only a few parenchymal CD3-positive cells (not shown) and CD20-positive cells could not be found (not shown). The Bielschowsky silver stain showed a rarefied axonal network, which is, however, not wholly lost (Fig. 8). In line with that, amyloid precursor protein (APP) staining marked discrete axonal damage throughout the lesions (not shown).

In summary, the hypercellularity at the lesion site was predominantly formed by macrophages, and the presence of macrophages with LFB and MBP-positive inclusions led to the staging of the lesion as active demyelinating, according to Van Der Valk and De Groot [9]. Hypercellularity was more pronounced at the lesion rim, a sharply defined lesion margin existed, and myelin protein phagocytic macrophages were present but sparce indicating a later phase of an active demyelinating stage. Such lesions are well compatible with the diagnosis of MS if appropriate clinical symptoms exist. A reference examination at the Institute for Neuropathology in Göttingen (Germany) independently confirmed the diagnosis.

## Diagnosis

### Multiple Sclerosis

Demyelinating processes can have several causes and can therefore be subdivided by their pathogenic mechanisms. Here, primary inflammatory demyelinating diseases represent the most common group [10]. Besides MS, acute disseminated encephalomyelitis (ADEM) and neuromyelitis optica (NMO) fall into this group. On the histopathological level, sleeves of demyelination that are usually found around small veins and venules characterize ADEM. These foci are associated with predominantly macrophages, but also lymphocytes and occasionally plasma cells are seen. In some cases, small perivascular hemorrhages are reported [11]. The ADEM affects mainly children and adolescents and typically occurs parainfectiously. Lesions usually appear bilaterally and asymmetric and are primarily found in the cortical white matter.

In contrast, NMO causes substantial demyelination of the spinal cord and optic nerve. Especially in the early stages, the brain stays unaffected [12]. Histopathologically, the spinal cord appears swollen, and massive macrophage infiltration, extensive loss of myelination, and axons followed by gray and white matter necrosis can be observed. Blood vessels become prominent and hyalinized. In a chronic state, the lesions appear gliotic, cystic, and atrophic [13]; however, classical plaques of demyelination in multiple sclerosis are often located in the periventricular white matter, spinal cord, and optic tracts. They can be classified according to the macrophage density and distribution pattern and myelin products phagocytosed by the macrophages within the lesion side. In an active demyelinating lesion, macrophages are dense and uniformly distributed all over the lesion and contain LFB or MBP-positive inclusions. Active but not demyelinating plaques are characterized by the absence of LFB or MBP-positive macrophage inclusions. Chronic active lesions typically show well-defined margins with a hypocellular center and a hypercellular rim, whereas chronic inactive lesions are defined as hypocellular throughout [9, 14, 15]. Taking all the histopathological characteristics of these primary inflammatory demyelinating diseases into account, an active demyelinating lesion seems most likely in the present case.

Further differential diagnoses of demyelinating processes comprise secondary causes, such as viral demyelination, acquired metabolic demyelination, hypoxic ischemic demyelination, or demyelination in the context of systemic diseases [10]. The latter incorporates, for instance, small vessel vasculitides. Due to appropriate clinical symptoms, such as subacute hearing loss, a severe disruption of the blood-brain barrier, and an appropriate age and sex of the patient, the attending physicians discussed a small vessel disease in terms of a Susac syndrome. In the specimen, perivascular CD3-positive T‑cell infiltrates can be detected. In contrast to the massive distinct demyelination, the axonal damage at the demyelinated sites is relatively sparce. These findings point to a primary cause of the demyelination and less to a secondary cause. Moreover, the typical characteristics of Susac syndrome, such as infarctions and thick-walled hyalinized vessels, are missing [16].
